# **“**Objectively terrifying”: a qualitative study of youth’s experiences of transitions out of child and adolescent mental health services at age 18

**DOI:** 10.1186/s12888-020-02516-0

**Published:** 2020-04-03

**Authors:** Kristin Cleverley, Lindsey Lenters, Emma McCann

**Affiliations:** 1 Lawrence S. Bloomberg Faculty of Nursing, University of Toronto, 155 College Suite, Suite 130, Toronto, M5T 1P8 Canada; 2 Margaret and Wallace McCain Centre for Child Youth and Family Mental Health, Campbell Family Mental Health Research Institute, Centre for Addiction and Mental Health, 80 Workman Way, Toronto, Canada

**Keywords:** Health transition, Transition to adult care, Participatory research, Thematic analysis, Child and adolescent psychiatry, Mental health services

## Abstract

**Background:**

Mental health issues presenting in childhood often persist into adulthood, usually requiring youth to transition from child and adolescent mental health services to adult mental health services at 18 years. Discontinuity of care during this transition period is well-documented and can leave youth vulnerable to adverse mental health outcomes. There is growing recognition of the need to improve transition-related care for youth leaving the child and adolescent mental health system. However, the perspectives and experiences of youth have not always been forefront in these discussions, and in particular, the perspectives of youth in the pre-transition period. This study qualitatively explores transition-related knowledge and experiences of youth both prior-to and after transition.

**Methods:**

A purposive sample of youth aged 16–19 years was recruited from two child and adolescent mental health programs. Youth were enrolled as part of a longitudinal follow-up study and had the opportunity to opt into this study. Interviews were transcribed and coded using NVivo11 software. Main themes were distilled through descriptive analysis following the principles of directed content analysis. The study followed the principles of participatory action research, engaging youth with lived experience navigating transitions in each stage of the study.

**Results:**

In-depth, semi-structured interviews were conducted with 14 pre-transition and 8 post-transition youth. All youth reported having either a mood and/or anxiety disorder for which the majority were receiving treatment at the time of the interview. The participants’ experiences were distilled into six major themes. Youth advocated for being considered partners in transition planning and to have increased control over transition-related decisions. Youth also made specific recommendations on how to improve continuity of care during the transition process.

**Conclusions:**

Transition planning should be individualized for each youth based on their developmental needs, transition readiness and ongoing mental health needs. Transition pathways, co-designed with youth and caregivers, should be developed to guide providers in transition best practices. Obtaining both the pre- and post-transition experiences of youth is crucial for developing a more complete of understanding of youth perspectives and implementing guidelines that improve transition quality and experiences.

## Background

As many as 1 in 5 children experience at least one mental health problem before the age 18 [1–3] with up to 70% of child and adolescent onset mental disorders persisting into adulthood [4]. This results in a high percentage of youth with mental illness who need to transition into adult services when they reach the child and adolescent mental health services (CAMHS) boundary, typically at age 18. Unfortunately, up to 60% of youth experience discontinuity of mental care during transition from CAMHS to adult mental health services (AMHS) [5, 6]. This discontinuity is not surprising given that the CAMHS to AMHS transition is fragmented and lacks effective evidence-based interventions [7–9]. This health services transition also occurs at a particularly vulnerable developmental stage, when youth are also simultaneously navigating social, family and academic life transitions [6, 10, 11]. The added complexity of the multiple concurrent life transitions has been reported by youth as a major obstacle in their ability to maintain continuity of mental health care when they transition out of CAMHS at age 18 [12].

Health services and policy makers from countries around the world (i.e. Canada, United Kingdom, United States, Australia, among others) are increasingly recognizing the importance of understanding and improving transitions from CAMHS to AMHS and developing effective interventions and models of care that are youth-informed and oriented [7, 13–18]. One of the most effective methods of informing models of care for transitioning youth is to learn from the lived experiences of youth who are transitioning from CAMHS to AMHS [19]. For example, previous qualitative research with youth report they perceive the following as enablers to more effective CAMHS to AMHS transitions: informal and gradual preparation for transition to AMHS, accessible and developmentally appropriate services, transfer planning meetings, periods of parallel care, consistency in key-clinicians, the involvement of parents, and individualized wrap-around services (intensive, individualized care planning and management in the community) [9, 12, 20–23].

A systematic literature review by Broad and colleagues [12] synthesized 18 qualitative research studies representing 253 service-users experiences of CAMHS to AMHS transitions across Sweden, the United Kingdom and the United States. The authors reported a lack of research that included the perspectives of youth who may have disengaged from mental health services once they left CAMHS. Suggesting the need to engage youth prior to transitioning out of CAMHS at age 18 and soliciting their perspectives regardless of whether they have successfully transitioned to AMHS services. This also acknowledges the fact that not all youth who reach the CAMHS service boundary at age 18 may require, or be eligible, for AMHS, an important discussion raised by Schraeder and Reid [24]. Service pathways out of CAMHS at age 18 are diverse, as highlighted by Munson and Colleagues in their mental health services utilization framework [25]. Research has consistently shown that less than 50% of youth transitioning directly from CAMHS to AMHS [5, 6, 26]. Transition experiences of youth may be different based on their service pathway when they leave CAMHS care at age 18 [12]. As such, there is a need to recruit youth prior to reaching the CAMHS service boundary at age 18, with interviews taking place with youth prior to and after exiting CAMHS to better understand the diversity of youth’s perspectives [12, 27].

Our primary aim is to qualitatively explore the experiences of youth in relation to their knowledge, expectations and experiences transitioning out of CAMHS services at age 18. Participatory action research [28, 29] will be utilized at all stages of this study, to ensure the unique expertise of youth who have experienced the mental health system is incorporated.

## Methods

### Setting and participants

Participants were identified through two participating CAMHS services. Both CAMHS services provide mental health care to children and adolescents up to age 18 with predominately mood, anxiety and to a lesser extent other disorders (i.e. behavioural, neurodevelopmental). Both CAMHS services are located in a large urban city (Toronto, Ontario) and both provided outpatient mental health care to children and youth aged 0–18 years. To be eligible, youth must be currently attending CAMHS treatment and aged 16–18 years (pre-transition group); or have attended CAMHS in the past year and had transitioned into AMHS at or around age 18 (post-transition group). The recruitment procedure employed at both CAMHS services involved identifying eligible youth for both the pre- and post-transition groups from lists of youth who were involved in the Longitudinal Youth in Transition Study (LYiTS) in Toronto, Canada [30]. Youth were classified as pre−/post-transition based on whether they were still receiving CAMHS services (pre) or had already transitioned from CAMHS into AMHS (post). LYiTS is a 5-year prospective cohort study tracking youth aged 16–18 as they transition out of CAMHS at age 18. Youth were approached by a research assistant after participating in LYiTS and asked if they would be willing to participate in a qualitative study of their transition experiences. In total, 17 youth were approached for pre-transition interviews, 14 agreed to participate. Another 8 youth were approached for post-transition interviews, of which all agreed to participate. Recruitment occurred over an 18-month period. The study was approved by both the University of Toronto and Centre for Addiction and Mental Health research ethics boards.

### Consent and interview procedure

Prior to conducting the interviews consent was obtained and the research assistant (RA) reviewed the parameters of confidentiality. Once consent was obtained the RA scheduled an interview at the CAMHS site at a time that was convenient for the youth. The interviews were conducted by either the first author (KC) or a master’s-prepared graduate student. The interviews were 30–60 min in length. A semi-structured interview guide was established to provide standardization of broad questions asked in both the pre- and post-transition interviews. The pre-transition (Group 1) interview questions focused on understanding the overall experience of entry into CAMHS and any transition planning that has been discussed with their clinical team. The post-transition (Group 2) interview focused on the transition out of CAMHS and the transition planning that occurred (or did not) with CAMHS and AMHS (where applicable) service providers. Sample question from the interview guides included; Pre-transition (a) “What supports or information would you want to have about this eventual change in mental health care?” and, (b) “What would a successful transition from CAMHS to AMHS look like to you?”; Post-transition (a) “Tell me about how (CAMHS clinic) prepared you for coming to AMHS?” and, (b) “Can you describe the similarities and differences between CAMHS and AMHS?”. The complete pre- and post- transition interview guides are available via the study contact author. We used extensive probing throughout the interviews to further understand the responses provided by youth during the interviews. Youth in the pre- and post-transition groups were asked to provide recommendations regarding how their transition experience could be improved.

This study followed participatory action research (PAR) methodology, with engagement of the youth guided specifically by the McCain Model for Youth Engagement [28, 29, 31, 32]. Two youth with lived experience navigating CAMHS to AMHS transitions reviewed and provided feedback on the semi-structured interview guides to ensure that it reflected transition experiences. They also reviewed the interview guides for terminology, simplifying language where needed. Lastly, one youth with lived experience (EM) participated in the analysis and write-up, as described below.

Qualitative interviews were auto-taped, anonymized, and transcribed verbatim. Transcripts were independently reviewed by two study researchers (KC and LL). The analysis followed the process recommended by Hsieh and Shannon [33] for directed content analysis, utilizing the process for directed content analysis described by Assarroudi and colleagues [34]. Utilizing a naturalist paradigm, directed content analysis is used to interpret meaning from text data in cases where research on a phenomenon already exists and would benefit from further description.

All interviews were read twice in full and a preliminary coding framework was developed. A subset of four interviews were independently coded using NVivo 11 [35] by two team members and inter-rater reliability was checked to ensure consistent use of the coding framework. Where differences were identified, the research team met to discuss these differences, clarifying the definitions and usage of codes, and adjusting the framework through consensus. The remaining interviews were coded in NVivo 11 by one researcher (LL). NVivo 11 was used to query the coded interviews, exploring combinations of codes to develop a deeper understanding of the source material. Memoing was used to guide and reflect on the authors’ evolving understanding of the major themes and weekly team meetings were used to collectively explore theme development. Further queries were run in NVivo to examine assumptions and check for accuracy of the team’s understanding, until a final set of themes was determined through consensus. An audit trail was maintained to document decisions made throughout the analysis process.

Within the PAR model, one of the youths with lived experience navigating CAMHS to AMHS transitions (EM) participated in the analysis phase of the project as a member of the research team, reviewing the initial coding themes and coding framework. Minor edits to wording and terminology of the themes were suggested by the youth. One youth (EM) also participated in the framing of the recommendations made by youth during the interviews, including suggesting minor edits to the terms used and the organization of the recommendations. This same youth (EM) then fully participated in the writing and revisions of this manuscript.

## Results

### Participant characteristics

There were 21 youth in the study, of which 13 were interviewed pre-transition, 7 were interviewed post-transition and 1 youth was interviewed both pre- and post-transition (see Table 1). Participants were between 16 and 19 years of age (mean 17.5, SD 0.91), all reported having either a mood and/or anxiety disorder, and all were in school or working at the time of the interview. Almost all youth (67%) identified as female and self-identified their cultural background as White (−European or -Canadian; 62%). All but one post-transition youth were currently utilizing mental health services.

**Table 1:** Participant Demographics and Transition Stage

Participant Number	Age	Currently in Mental Health Services (yes/no)	Transition Stage (pre/post)
Participant #1	18	Yes	Pre-transition
Participant #2	18	Yes	Pre-transition
Participant #3	16	Yes	Pre-transition
Participant #4	18	Yes	Pre-transition
Participant #5	17	Yes	Pre-transition
Participant #6*	18	Yes	Pre-transition
Participant #7	17	Yes	Pre-transition
Participant #8	17	Yes	Pre-transition
Participant #9	16	Yes	Pre-transition
Participant #10	17	Yes	Pre-transition
Participant #11*	18	Yes	Post-transition
Participant #12	19	Yes	Post-transition
Participant #13	18	Yes	Post-transition
Participant #14	18	Yes	Post-transition
Participant #15	19	Yes	Post-transition
Participant #16	19	Yes	Post-transition
Participant #17	18	Yes	Post-transition
Participant #18	16	Yes	Pre-transition
Participant #19	17	Yes	Pre-transition
Participant #20	17	Yes	Pre-transition
Participant #21	17	Yes	Pre-transition
Participant #22	18	No	Post-transition

*Participants #6 and #11 are the same individual interviewed 8 months apart, pre and post transition

### Themes

Pre- and post-transition youth discussed a range of experiences, which fell into six main themes described below. Overall, there was alignment in the ways that pre- and post-transition youth spoke about transition, with post-transition youth drawing on lived experience and pre-transition youth drawing on anticipatory thinking. In other words, their expectations of the transition process were likely informed by their experiences of CAMHS to-date as well as their general life experience. Due to the complimentary nature of themes arising from pre- and post-transition youth, the insights are integrated with key differences highlighted throughout.

Youth also provided recommendations for improving the transition process, some of which are aimed at the health care system and health care providers, and others directed towards youth who will be transitioning out of CAMHS at age 18.

### Shifting awareness of the meaning of ‘transition’

Participants were largely aware of the need to transition out of CAMHS as a general concept. As articulated by one participant, “it was always in the back of my mind because I was seeing someone that was meant for younger people and I was almost turning 18” [Participant 16 (Post-transition, aged 19)]. However, this awareness did not seem to hold personal meaning until transition was more imminent. Several participants described the moment when their awareness of the need to transition took on personal meaning: “I had known about it but it was more of an “aha” moment. I was just like oh I’m going to/I can’t like, look for youth [services] anymore because I’m technically not youth, like a lot of psychiatrists stop at eighteen and a lot of them start at eighteen. So like, it’s difficult.” [Participant 19 (Pre-transition, age 17)].

Many participants reported feeling nervous when the notion of ‘transition’ shifted from an abstract concept to a concrete inevitability. Reflecting on a conversation with their provider about transition, one participant said, “it’s like, ‘oh, you’re going to transition, and it will be fine’ but, like, will it? Because I don’t know how to do it. It’s just hard” [Participant 13, (post-transition, age 18)]. Contemplating the implications of transition for the first time brought up uncertainties and unanswered questions for many participants, for example around whether or not mental health services would be covered by their health insurance: “I get a little nervous because I’m not sure if I would have to start paying any fees for additional treatment if I transition into treatment for adults” [Participant 1 (Pre-transition, age 18)].

### Ready or not to transition

Participants described a range of feelings related to transition readiness. For many, ‘transition’ meant wrapping up treatment with the CAMHS clinician and having their prescriptions transferred over to their primary care provider. In these cases, the participants described feeling ready to transition out of CAMHS. One participant who reported feeling ready to transition described their situation as follows, “I stopped [receiving services], I think about a year ago. Because I started meds and then I stopped really needing the therapy” [Participant 14 (Post-transition, age 18)]. This participant reported that it was “completely my decision” to taper off therapy. Other participants described not feeling ready to transition due to uncertainty about, and feeling underprepared for, the next stage of their care: “I was not ready for the last day. I wasn’t ready to leave because I did not know what would happen when I left” [Participant 17 (Post-transition, age 18)].

When asked about readiness to transition, there was an element of resignation in many participants’ responses. This was particularly evident among participants in programs with strict eligibility criteria: “Well, I’m going to be discharged whether I’m ready or not” [Participant 21 (Pre-transition, age 17)]. Other participants explained that they did not feel involved enough in transition-related decisions to be able to formulate opinions about their care. As explained by one participant, “I’m not sure how I feel. I don’t really know about [the AMHS referral] enough to think much about it” [Participant 19, (Pre-transition, age 17)].

Interestingly, one post-transition participant reflected on how they were simultaneously ready and not ready to transition, due to the multi-dimensional nature of their treatment: “I was ready but I wasn’t ready… I still wanted to see the psychiatrist because he was going to eventually take me off the medication and that is not happening now with my family doctor. So I wasn’t ready because of that, but I was ready because I wasn’t doing counselling anymore, I did not really need it. I was, like, finally doing well” [Participant 12 (Post-transition, age 19)].

### Mixed reactions to transitional age of 18 years

Participants expressed dual feelings about the transition age being set at 18 years. On the one hand, it made sense to them because it aligned with the age that they needed to transition from a pediatrician to a primary care provider. On the other hand, participants expressed a belief that 18 years is an arbitrary cut-off, and many wondered why there was not more flexibility and individualization of transition age. Many participants felt that if there had to be an age cut-off it should be early 20s, when youth’s lives are “more stable” [Participant 15 (Post-transition, age 19)] and they have become “fully adult” [Participant 13 (Post-transition, age 18)].

Pre-transition participants also expressed feeling like they were in an awkward in-between stage. On the one hand they felt out of place in CAMHS: “It was very obvious that I was in a child service. Like at one point there was a six-year-old on the unit… and I turned 18 in the unit so I was always the oldest person there” [Participant 17 (Post-transition, age 18)]. On the other hand, they felt equally concerned about the prospect of receiving adult-oriented services and expressed worry about being treated by clinicians who don’t specialize in youth mental health. As expressed by one participant, “[the transition to AMHS] will bother me because I’ll be like having the same therapy for my brain as 60s and 70s years old” [Participant 20 (Pre-transition, age 17)].

### Lack of information, preparation and involvement in the transition planning process

Overwhelmingly, pre-transition participants expressed feeling uninformed, disconnected and confused about the transition process. Even participants who reported that someone on their care team had mentioned transition were unsure of how the process would unfold: “well I mean, they like tell me, like, I’m going to have to leave, but like… I really don’t know.” [Participant 19 (Pre-transition, age 17)]. Pre-transition participants also described feeling unsure about AMHS options: “when I think about it right now, I don’t even know where to look for adult services” [Participant 1 (Pre-transition, age 18)].

Post-transition participants similarly spoke about feeling unprepared, uninformed and uninvolved in the transition planning and execution. For example, one post-transition participant described their transition meeting as vague, with little concrete information or direction given: “They did not necessarily point me in any direction and say ‘this is the right path for you’, but they did say ‘you need to continue to see a psychiatrist and a psychologist’” [Participant 17 (Post-transition, age 18)].

Post-transition participants also described having very few opportunities to be engaged in transition planning and being given very little choice regarding referrals. One participant described being told “we are going to give this one a go”, rather than being given options [Participant 15 (Post-transition, age 19)]. This experience was echoed by another post-transition participant who was transitioned to a more specialized service, with little consultation or explanation. “[The CAMHS program] said they couldn’t be a support… they told me a couple months in advance, they just forwarded me out… there really wasn’t anything in place, they did not really explain to me the program that I was going to at [the new hospital]” [Participant 17 (Post-transition, age 18)].

### Confusion around roles and responsibilities within the transition process

Participants spent considerable time during the interviews talking about their experience of transitioning to adulthood, and the thoughts and feelings related to ‘growing up’ – they expressed feeling that they ought to be taking on more responsibility for their own health, and that they desired more independence and autonomy while simultaneously expressing the need for continued support from their caregivers. As one participant put it, “I have been insisting to my mom recently that obviously I do need help but I am allowed to make my own decisions now legally” [Participant 13 (Post-transition, age 18)].

Importantly, many pre-transition participants expressed confusion around not knowing who was meant to be coordinating the transition process, and they expressed a strong desire for someone knowledgeable and trustworthy to take responsibility for their care coordination. The transition out of CAMHS at age 18 was described as an intimidating process, particularly with the myriad of other life changes happening at this stage of life: “We’re already scared enough as is. You really need to stop scaring us so much. When the system doesn’t seem to have any plan, it’s objectively terrifying” [Participant 3 (Pre-transition, age 16)].

Some post-transition participants described being left to coordinate their transition to AMHS on their own, which was difficult given the opaqueness of the system. “[My provider] had just told me ‘just look into counselling and schedule something as soon as you get to University’. But I didn’t. I was in-between healthcare providers at that time and I did not have any back-up people to kind of go talk to” [Participant 13 (Post-transition, age 18)]. Even though many post-transition participants reported they had had conversations with their CAMHS clinicians about transitioning, they had not expected to receive so little concrete support to ensure an appropriate transition actually took place.

Some participants had more positive experiences with their mental health care providers, reporting that someone within the system took the lead role coordinating the transition process. In one interview, a participant described being connected to a family mental health service whose purpose is to help navigate referrals. However, this participant articulated that the service would have been more helpful if they had been connected earlier [Participant 11 (Pre-transition, age 18)]. Another participant described unexpected levels of support: “I thought it would just mean like that is it, no more, nothing else. But um the psychiatrist really worked with me to try and get my family doctor to uh take things now” [Participant 12 (Post-transition, age 19)]. Another post-transition participant mentioned that a CAMHS nurse had accompanied them to their AMHS intake appointment, which the participant described as helpful yet bittersweet because they had not been given a choice of AMHS providers [Participant 15 (Post-transition, age 19)].

### Concern over transition gaps leading to poor mental health outcomes

Pre-transition participants who believed they would continue to need mental health services described feeling concerned about potential gaps in services during transition from CAMHS to AMHS. They were concerned about the potential impact on their mental health. As expressed by one youth “I’ll crash and burn and I don’t know what that will look like at this point but I kind of think it’s inevitable if I don’t figure [my transition] out” [Participant 3 (Pre-transition, age 16)].

Indeed, post-transition participants found that this was often the reality – there was a lack of seamlessness in their transition from CAMHS to AMHS, which in some cases had a negative impact on their mental health. This is exemplified in one participant’s experience leaving CAMHS with no referral or support to find appropriate AMHS: “I am a very anxious person. So kind of starting new things is really hard for me. Especially being expected to do so alone a lot of the time. Which I guess it is a big part of why I ended up in the hospital when I did” [Participant 13 (Post-transition, age 18)]. The repercussions of gaps in mental health care is further exemplified by one participant’s account: “There’s a lot of physical stress trying to find [a new provider]. What I am dealing with crippled me. I can’t go to school, [it’s] hard to wake up, hard to eat…” [Participant 11 (Post-transition, age 18)].

## Recommendations

Overall, post-transition participants offered more detailed and specific recommendations for how to improve the transition process, as compared to pre-transition participants, an expected finding given that post-transition youth were drawing from lived experience. Pre-transition participants tended to focus more on concerns over the loss of relational continuity with their CAMHS providers. Participant’s recommendations were mainly directed towards the mental health system, specifically their expectations of their CAMHS clinician and program. Although not a primary focus, some participants made specific suggestions for other youth and their families related to the transition process. Table 2 provides a summary of all recommendations given by the participants along with illustrative quotes.

**Table 2:** Youth Recommendations for Improving Transition Experiences

**Recommendations for the Health Care System and Health Care Professionals**	
Partner with youth in transition planning	• Offer youth more choice and control around transition-related decision-making (i.e., around parental involvement, AMHS referral, gender of AMHS clinician) • Create individualized transition plans
Timing of transition is purposeful	• Base transition timing on developmental and clinical readiness, not age • Discussion transition with youth early, well in advance of transition date • Ensure all stakeholders (i.e. youth, caregivers, CAMHS, AMHS, transition support workers/navigators) are involved early in the transition process
Ensure access to transition-related information	• Provide details about what to expect during the transition process • Provide information about what to expect from AMHS (i.e., differences in cost). • Offer more ways for youth to get to know AMHS options and providers in advance (i.e. Print resources, “open house” to meet AMHS providers). • Provide youth with access to information related to their plan of care, including any referrals that have been sent, key service contacts and next steps in their treatment plans, and ensure youth understand the information provided
Ensure relational continuity between CAMHS and AMHS	• Connect youth to AMHS before CAMHS discharge • Ensure primary CAMHS provider is involved in the transition process • Provide youth with support and information to identify appropriate AMHS • If youth are not referred directly to AMHS, provide the option to return to CAMHS for referral support later on, or the option to taper out of CAMHS services gradually. • Have communication between CAMHS and AMHS clinicians and ensure overlapping CAMHS and AMHS care • CAMHS should be responsible for following up to make sure that AMHS referral was appropriate and completed
Additional programmatic recommendations	• Offer CAMHS transition support groups • Create dedicated mental health services for transition aged youth • Recognize the transition milestone (i.e., organize a celebration to mark CAMHS discharge)
**Recommendations for Other Youth and Their Families**	
	• Manage expectations (be prepared for the process to confusing) • Ask questions as soon as you have them • Don’t hesitate to reach out to your personal support network during the transition process • Recognize that it takes time to get connected to appropriate AMHS; start early

Overwhelmingly, participants asked to be considered as partners in the transition planning process; to have more choice in transition-related decision-making. At the same time, participants also expressed the need for more support from experts who know how to navigate AMHS. Additionally, participants expressed that they want transition planning to begin earlier. “I wish someone had told me way sooner, like much more in advance, about what would happen. Like go through it more as opposed to just one day having the forms and signing them and then, like kind of being in the dark until then” [Participant 17 (Post-transition, age 18)]. Youth also expressed that transition planning should be provided consistently to all youth: “Even if you feel like you don’t need [transition planning], I feel like it should still be offered and offered early. Like, before you are about to turn 18 for sure” [Participant 15 (post transition, age 19)]. Participants also asked for a more flexible transition age, based on clinical and developmental readiness, rather than a rigid age cut-off: “I don’t think there should be an age [cut-off] at all. I think it should be when you’re ready” [Participant 7 (Pre-transition, age 17)].

Participants called for greater access to information, both about what to expect from the transition process as well as the range of potential AMHS options. For example, one participant called for “more information about how therapy works outside of being like under 18” [Participant 13 (Post-transition, age 18)], particularly in light of differences related to coverage of services. Additionally, participants noted that it was challenging to recall important details after appointments and therefore recommended providing physical copies of information.

Participants also called for greater continuity of care between CAMHS and AHMS. This included recommendations around ensuring seamless transfer of files, opportunities for CAMHS and AMHS clinicians to hold at a joint meeting, and allowing for a period of parallel care while youth adjust to the new AMHS setting. Participants also spoke about the importance of knowing where to seek services later on, if they weren’t being transferred to AMHS. As explained by one post-transition participant “I am feeling a lot better but what happens in a few months if I am not? What can I do? Because I don’t know what I should do now if I need someone…” [Participant 12 (Post-transition, age 19)].

Several post-transition participants offered advice for other youth who are transitioning and their families, including expecting the process to be confusing and difficult, reaching out to support networks, and starting the process early to avoid a gap in services. “Ask as many questions as you can. Everything that comes to your mind… say it now or forever hold your peace” [Participant 17 (Post-transition, age 18)].

## Discussion

The objective of this paper was to explore the experiences of youth who are transitioning out of CAMHS at age 18 and those who have recently transitioned out of CAMHS at age 18. Themes emerging from the data include: (1) shifting awareness of the meaning of ‘transition’; (2) ready or not to transition; (3) mixed reactions to transition at age of 18 years; (4) lack of information, preparation and involvement in the transition planning process; (5) confusion around the roles and responsibilities within the transition process; and, (6) concern over transition leading to gaps in mental health services. While there was some congruency within both the pre- and post-transition interviews, there were important differences that continue to support the importance of interviewing youth in various stages of the transition process [12, 27]. Pre-transition youth provided nuanced insight into gaps in the system, drawing on their immersion within the transition experience. Whereas post-transition youth, having been through the experience, were better equipped to provide concrete recommendations on improving transition outcomes. Thus, this study contributes additional richness to the existing body of literature through a dual focus on pre- and post-transition perspectives.

Both the pre- and post-transition youth in this study advocated for earlier and more detailed preparation for the transition out of CAMHS at age 18. Youth described a shifting awareness of their need to leave CAMHS at age 18, in other words, their relationship to transition changed as they got closer to the transition period. It was not surprising then that youth in this study reported differing degrees of knowledge about the need to transition out of CAMHS at age 18. This may be related in part to the degree of knowledge of a discharge or transition plan. It is difficult to know whether this planning is simply not happening, or whether it’s happening in a way that is not visible or not digestible to youth. Either way, youth aren’t benefiting from the security of having a transition plan.

Youths’ varying feelings of readiness towards transition may also be related to their perception or understanding of their need for ongoing mental health care. This is an important finding because, as Schraeder and Reid [24] point out, youth who are asymptomatic prior to transfer may disengage from the transition process, particularly if the transition process is poorly managed. This can leave youth vulnerable to adverse outcomes should they experience a recurrence later on, without having supports in place. Schraeder and Reid [24] argue for a more streamlined transition process that lowers the risk of youth disengaging, and they also argue for clear, evidence-based guidelines around who should transfer.

This research also highlights that transitions between CAMHS and AMHS (or ongoing mental health care) need to be patient-centred, developmentally friendly, and consider the social support and service-needs of the youth. Developmentally not all youth may be ready to take over all decision-making, particularly if, like youth in this study, they are not aware of the care options in AMHS or their mental health care needs. As such, providing pathways that highlight freedom of choice to youth and their caregivers within a structure of support from clinicians and providers with whom they trust is ideal. In this study, that youth had mixed reactions to need to transition out of CAMHS at age 18, some youth reporting feeling ready, while others thought allowing for more flexibility in the age would be beneficial. This coincides with the emerging literature and clinical best practice of youth specific mental health clinics, where the age 18 transition for CAMHS has been increased to 24 or 25 years of age, reported to be a more developmentally appropriate age to transition to AMHS [36–38].

Based on the findings from this study, transition guidelines should be flexible and individualized, and should involve youth (and their caregivers) as partners in decision-making early in the process. The development and implementation of service pathways or guidelines to support the transition of youth out of CAMHS would also provide much-needed guidance to clinicians about the transition process, options for transition pathways, and highlight best practices in ensuring continuity of care [6, 39]. These guidelines should be co-designed with youth, caregivers and clinicians, and be informed by best available evidence to support successful transitions in care. A recent scoping review identified 26 core components of successful CAMHS to AMHS transitions that could inform the basis for transition pathways or guidelines [40].

Throughout their interviews youth describe not understanding who was coordinating their care, particularly as they transition out of CAMHS – several noted that they felt unsupported in their transition. From a mental health systems perspective, this means ensuring that prior to leaving CAMHS, youth ought to have access to basic information regarding where they can access mental health care, including who they could contact should they need care particularly if there is a wait or lag in uptake into AMHS. Ensuring youth are equipped with this information may reduce the potential impact of poor transitions or the stress associated with not knowing where to access mental health care. In this study, post-transition youth describe stress and distress related to having undergone a poor transition process or not being engaged in the transition discussion. Research has shown that youth who have a poor transition out of CAMHS often experience improper medication monitoring, may re-emerge later in crisis and are at increased risk for serious and enduring mental health problems [6, 41–43]. Transition guidelines and pathways should then include the ways in which youth can re-engage in the mental health system should they experience a poor transition out of CAMHS.

Incorporating youths’ recommendations for improved CAMHS to AMHS transitions in care is an important aspect of ensuring the knowledge and experiences of youths who have transitioned contributes to improvements in current transition practices and policies. Broad et al. [12] reported on youth recommendations for positive transition experiences as part of a qualitative literature review. The authors categorized the recommendations according to pre- pre- and post-transition periods. Their recommendations overlap with the findings in this study, including notions of promoting individualized care plans and flexible approaches, involving youth as partners and supporting autonomous decision-making, and ensuring adequate support and continuity of care [12]. One notable difference is that Broad et al. [12] report that youth recommended physical care environments geared towards youth, whereas recommendations related to the physical care environment did not emerge in this study.

This research project utilized the principals of participatory action research (PAR) by engaging youth with lived experience of navigating transitions from CAMHS to AMHS. This collaboration was guided by the McCain Model for Youth Engagement [32]. As noted in previous research [28, 29, 31, 32], the involvement of youth with lived experience strengthened this project in several important ways. Notably, by collaborating in the design of the study and then in the analysis and interpretation of results. The engagement of youth ensured the research questions were timely and relevant to the population being interviewed, likely improving the quality of the responses from youth. The results of this PAR study can be utilized in the co-design and co-production of transition guidelines or pathways that support continuity of mental health care for youth leaving CAMHS at age 18. The collaboration with youth with lived experience in the co-design of transition programs is emerging as a best practice in mental health care [44].

When interpreting the results, it is important to note that this study captured general pre- and post-transition experiences, rather than within-person changes. This was due to timing constraints that did not allow for all youth to be interviewed pre- and post-transition. Future studies could consider a study design that explores within-individual experiences pre- and post-transition. Additionally, the sample included more pre-transition youth and fewer post-transition youth. However, it is important to note that no new themes emerged during the final post-transition interviews.

Similar to all qualitative research, the findings are intimately tied to context. Thus, practitioners should independently evaluate the applicability to their specific contexts. For example, this research was conducted at two CMAHS that predominately treat youth presenting with depression and/or anxiety and in a major Canadian city where multiple specialty mental health service agencies operate. Caution should be exercised when applying the findings to a context with other diagnosis (i.e. psychosis) and different access to specialty mental health services. Additionally, interviews were conducted at the CAMHS agencies where participants were receiving services, which may have influenced participants’ comfort level with openly sharing their experiences of care. The potential for negative impact on data quality was minimized by conducting the interviews in non-clinical areas by interviewers who were not involved in participants’ care; however, future research might want to consider conducting interviews outside of the CAMHS agencies. Lastly, while these findings may have applicability to a variety of regions around the world, the type of health care system and how mental health services are accessed is unique in different countries. As such, studies on the youths’ expectations and experiences of transitions from CAMHS to AMHS, need to be conducted in other parts of the world. Additionally, as pointed out by Cleverley and colleagues (2018) in their recent scoping review of processes that facilitate CAMHS to AMHS transitions, future research needs to focus on how the health care system, including funding models and access, impacts on the success of CAMHS to AMHS transitions experienced by youth [40].

## Conclusion

The findings from this qualitative study is based on the experiences of youth pre- and post-transition from CAMHS at the age of 18. The findings provide information on not only the type of information youth need prior to transitions, but also when and how they should receive this information. It underscores the importance of co-designing transition guidelines and pathways with youth that consider the developmental needs, transition readiness, and ongoing mental health needs of each youth. The results of this study also underscore the importance of including the perspectives of youth both pre- and post-transition to ensure the range of expectations versus reality are captured. Finally, it is hoped that the findings of this study will lead to the co-design of interventions with youth that improve transition quality and experiences.

## Data Availability

The qualitative data that supports the findings of this study are potentially identifiable and not therefore suitable for sharing via a public database. The data are however available from the authors upon reasonable request - subject to ethical permissions and participant consent.

## Abbreviations

AMHS: Adult mental health services
CAMHS: Child and adolescent mental health services
LYiTS: Longitudinal Youth in Transition Study
PAR: participatory action research
